# Antibiotic prophylaxis in pediatric urology

**DOI:** 10.4103/0970-1591.40605

**Published:** 2008

**Authors:** Seung-Hun Song, Kun Suk Kim

**Affiliations:** Department of Urology, CHA General Hospital, College of Medicine, Pochon CHA University, Seoul, Korea; 1 Department of Urology, Asan Medical Center, University of Ulsan College of Medicine, Seoul, Korea

**Keywords:** Antibiotic prophylaxis, child, urinary tract infections

## Abstract

Urinary tract infection (UTI) is a common problem in infants and children. Children at risk for UTI such as vesicoureteric reflux (VUR) commonly receive prophylactic antibiotics to prevent renal scarring, which may lead to complications such as hypertension or end-stage renal disease. Recurrent UTI, with or without VUR, is the most common reason for long-term antibiotic prophylaxis in infants and children. However, the efficacy and importance of long-term antibiotic prophylaxis have not been assessed in well-controlled, prospective studies. Nitrofurantoin, trimethoprim/sulfamethoxazole have been used as prophylactic antibiotics for the prevention of UTI in children. Such medications are mostly safe in children for the long-term prophylactic therapy. Serious side effects are extremely rare and most are reversible with discontinuation of therapy. Although it is difficult to perform prospective studies in children and many factors are involved in the clinical course and prognosis of these patients, further studies are needed to evaluate the actual benefits of prophylactic antibiotics. Meanwhile, in infants and children with risk factors, long-term antibiotic prophylaxis should be considered, at least until there is evidence that these patients are not endangered by avoiding it.

## INTRODUCTION

The widespread use of antenatal ultrasonography has markedly increased the detection of congenital urological anomalies, most commonly hydronephrosis and urologists treating pediatric patients have experienced a shift in treatment paradigms in the last decades. Newborns are infrequently treated with early surgery; rather, most are followed nonoperatively, with the goal of preserving renal function. During the follow-up period, urinary tract infection (UTI) deleterious to the kidney may develop. Thus, long-term prophylactic antibiotic are recommended to many children with urological diseases, such as reflux and megaureter. The aim of prophylactic antibiotic therapy is to provide sufficient level of antimicrobial agents in the bladder to prevent bacterial multiplication and the ideal prophylactic antibiotic provides high urinary antibiotic excretion with low serum and fecal concentrations. Historically, Smellie *et al.* and Lohr *et al.* evaluated the effectiveness of low-dose antibiotics for the prevention of UTI while on treatment.[1,2] Based on these early studies, the concept of antibiotic prophylaxis was adapted by many groups.[3,4] This review concentrates primarily on antibiotic prophylaxis in pediatric urology.

## URINARY TRACT INFECTIONS IN INFANTS AND CHILDREN

The traditional definition of clinically significant UTI has been ≥10^5^ colonies/ml for clean catch specimen, ≥5 × 10^4^ colonies/ml for catheterized specimens. The UTI is the second most common problem in infants and children, with approximately 1% of boys and 3-5% of girls suffering from at least one UTI during childhood.[5] Moreover, morbidity is not limited to the acute period of illness. In general, although infections of the bladder (cystitis) cause substantial morbidity, they are not considered as serious bacterial infections. In contrast, infections that involve the kidneys (pyelonephritis) may cause both acute morbidity and lead to scarring, resulting in hypertension and chronic renal disease. Permanent renal damage after acute pyelonephritis has been estimated to occur in 5-20% of children based on intravenous urography findings. Using DMSA scans, however, the incidence of renal scarring after acute pyelonephritis was much higher, reaching 40%.[6]

The incidence of scarring increases with each episode of pyelonephritis. Infants are more vulnerable than older children to the development of UTI.[7] The higher vulnerability of infant kidneys may partially be due to the fact recognition and treatment of pyelonephritis is often delayed within the first year of life. Recurrent UTIs are observed in 30-50% of children after the first UTI. Of these, approximately 90% occurs within 3 months of the initial episode.[8] The bacteria most frequently responsible are Gram-negative organisms, with *Escherichia coli* accounting for 80% of urinary tract pathogens. Because younger children and infants often have nonlocalizing symptoms with UTI, the physician must have a high index of suspicion. The basic aim of antibiotic prophylaxis in children with urinary tract anomalies and/or recurrent UTI is the reduction of UTI frequency.

## INDICATIONS AND DURATION OF ANTIBIOTIC PROPHYLAXIS

It is generally recommended that children who are considered to be at risk for recurrent UTIs and potential scarring should receive prophylactic treatment. The aim of prophylactic antibiotic therapy is to provide sufficient level of antimicrobial agents in the bladder to prevent bacterial multiplication and the ideal prophylactic antibiotic should provide high urinary antibiotic excretion with low serum and fecal levels. Recurrent UTI, with or without vesicoureteric reflux (VUR), is presently the most common reason for long-term antibiotic prophylaxis in infants and children. Neonates and infants <1 year of age who present with a febrile UTI are also candidates for antibiotic prophylaxis, because approximately one-third of these patients are at risk for symptomatic recurrences.[9] In UTI with obstructive lesions, prophylaxis is considered until the underlying lesion is successfully corrected or has diminished spontaneously.[10] With the increased detection rate of asymptomatic urinary malformations by sonographic pre- and postnatal screening, antibiotic prophylaxis has been recommended following the detection of even asymptomatic reflux or urinary tract obstruction.[11]

It is widely accepted that the risk for pyelonephritic renal scars decreases with increasing age. However, the duration of urinary tract antibiotic prophylaxis and the time for its discontinuation are still unclear. Prophylactic treatment is usually continued until the abnormal condition resolves; for example, the spontaneous resolution or surgical correction of VUR, obstruction. Recently, Al-Sayyad *et al.* suggested that prophylactic antibiotics could be safely discontinued in selected children.[12]

## BENEFITS OF ANTIBIOTIC PROPHYLAXIS

Prophylactic treatment consists of a single dose of drug at bedtime, so that the antibiotic can be retained all night in the bladder, thus enhancing its effectiveness. The dose of prophylactic antibiotics in infants is about 20-30% of the full adult dose. Historically, Smellie *et al.* and Lohr *et al.* evaluated the effectiveness of 2-6 months on low-dose antibiotics for the prevention of UTI while on treatment.[1,2] Based on these early studies, the concept of antibiotic prophylaxis was adapted by many groups. Smellie *et al.* recommended using antibiotic prophylaxis in children who have recurrent UTI.[3] They also used prophylaxis in children whose kidneys might be adversely affected by any recurrence of infection, especially young children with VUR, whether or not the kidneys were already scarred.

Vesicoureteric reflux is the most common indication of antibiotic prophylaxis in anticipation of spontaneous reflux regression. Prophylactic antibiotics for children with VUR have become an alternative to surgery for several decades, especially since the reports of the International Reflux Study Group.[13,14] Those studies showed no advantage of surgical correction over prophylactic antibiotics with respect to the occurrence of new renal scarring. Recently, endoscopic subureteric injection of various materials has become an alternative minimally invasive method of correcting VUR in children.[15] However, there is considerable disagreement regarding the best treatment for VUR. A meta-analysis of randomized controlled trials about antibiotic and surgery concluded that it is not clear whether any intervention for children with primary VUR does good than harm.[16]

There are different concepts regarding the prevention of UTIs in pediatric patients with congenital dilation of the upper urinary tract, such as those caused by ureteropelvic junction obstruction (UPJO) or megaureter. Some authors recommend prophylactic antibiotic to all infants until a furosemide renogram excludes significant obstruction or until surgical reconstruction has been performed, while others administer prophylactic antibiotics only for patients with significant dilated upper urinary tracts. In the study by Madden *et al.*, no difference was observed in the incidence of UTI between children who did and did not receive antibiotic prophylaxis (14% vs. 19%), suggesting that antibacterial prophylaxis in children with UPJO was not effective.[17] However, Song *et al.* reported a high incidence of UTI in patients with severe obstructive hydronephrosis followed without antibiotic prophylaxis and advocated antibiotic prophylaxis in obstructive hydronephrosis.[18] Unfortunately, the actual risk of UTI in these patients is not known, because no prospective, randomized studies have been performed.

Recently, the efficacy of antibiotic prophylaxis has been questioned in several reviews.[19,20] Most of the authors of these articles criticize the lack of evidence for using antibiotic prophylaxis, due to the low quality of clinical trials and the small number of patients. For example, there is considerable uncertainty about the effectiveness of long term, low dose antibiotic administrations for the prevention of UTI in children.[21] Moreover, in the study by Conway *et al.*, antibiotic prophylaxis was not associated with decreased with risk of recurrent UTI, but was associated with increased risk of resistant infections.[22] Although it is difficult to perform prospective studies in children and many factors are involved in the clinical course and prognosis of these patients, further studies are needed to evaluate the actual benefits of prophylactic antibiotics.

## CONGENITAL HYDRONEPHROSIS AND OBSTRUCTION

Hydronephrosis is the most common abnormal finding in pediatric urology associated with genitourinary abnormalities, being present in 50-87% of affected patients. During the last decade, the controversy surrounding postnatal management of prenatally diagnosed hydronephrosis has shifted toward an initial nonsurgical approach, even in patients with severe dilation, since most cases of congenital hydronephrosis resolve spontaneously. There have been several studies examining the true natural history of patients with asymptomatic congenital hydronephrosis.[23,24] These reports have shown no decline in function and occasionally an increase in relative function toward normal in the hydronephrotic kidney. During follow-up, however, some children with hydronephrosis develop significant uropathy and are therefore susceptible to UTI. In other children, the hydronephrosis is either very well tolerated, with no complications or resolves spontaneously. Our knowledge of the significance of congenital hydronephrosis is continuing to evolve and the importance of prophylactic antibiotics in infants with this condition has not been formally tested.

Pediatric urologists are basically interested in obstruction to prevent the clinical sequelae associated with it, including renal functional impairment, infection, stones, hypertension, and symptoms of pain or hematuria. Although hydronephrosis and obstruction are closely associated, hydronephrosis may occur in the absence of clinically significant obstruction. Initial management of congenital hydronephrosis is mostly conservative. It is due to variable progress from patient to patient and the technical difficulties about the studies, which are used for the estimation of the real obstruction. Medical management of reflux, hydronephrosis and dysfunctional voiding has allowed many children to avoid surgery. Therefore, pediatric urologist is now confronted with a wide spectrum of degrees of hydronephrosis and challenged to predict the children who may be at risk from whatever degree of obstruction is present. It is expected that an infection accompanied by ureteric obstruction can cause significant renal damage and the recognition and relief of the obstruction remain the primary concern of the urologist. Several studies showed that many neonatal kidneys with severe hydronephrosis, however, are not biologically obstructed despite even a profound initial decrease in renal function.[25,26] A continuing debate in the management of congenital hydronephrosis is concerned with the definition of when significant obstruction is present. Unfortunately, all of the standard tests used to assess hydronephrosis are inaccurate. The diagnosis in most cases is only possible by repeated investigations and changes of the variables during longer follow-up. Clinical judgment should remain the most important factor in the decision-making process in the care of congenital hydronephrosis and obstructive uropathy.

## VOIDING DYSFUNCTION AND UTI

Voiding dysfunction is a broad term indicating a voiding pattern abnormal for the child's age. Treatment of underlying voiding dysfunction and constipation is an important component of successful management of UTI in children. It has been hypothesized that UTI may develop in girls with voiding dysfunction due to the backflow of urine laden with urethral bacteria, a phenomenon that results from the voiding dysfunction. Conversely, many children with recurrent UTIs are found to have abnormal urodynamic studies even in the absence of any neurologic abnormalities.[27] In the study by Hellerstein and Nickel, voiding dysfunction has been shown to be an important risk factor for the recurrence of UTI.[28]

In neurogenic bladder, bacteriuria and pyuria, both asymptomatic and symptomatic, occur frequently. Infections of the lower urinary tract may lead to complications and morbidity affecting both the lower and upper tracts, including bladder and renal calculi and pyelonephritis. Therefore, preventing UTI in these patients has been an important concern. Johnson *et al.* reported a positive effect of antibiotic prophylaxis in pediatric patients with neurogenic bladder.[29] However, the efficacy of antibiotic prophylaxis has not been investigated sufficiently in patients with neurogenic bladder dysfunction. A recently published meta-analysis of 15 studies indicated that antibiotic prophylaxis did not significantly reduce symptomatic UTIs.[30]

Neonatal circumcision needs a brief comment. Uncircumcised male infants are known to have a greater risk of developing UTI during the first year of life. Many studies have reported the protective effect of neonatal circumcision against UTI and most complications of circumcision are minor, occurring at a rate of about 2%.[31] However, it is generally accepted that there is insufficient evidence to recommend routine neonatal circumcision. A recent systemic review suggested that the net clinical benefits of circumcision is likely only in boys at high risk of UTI considering the benefits and complications of circumcision.[32]

## SAFETY AND ADVERSE REACTIONS OF PROPHYLACTIC ANTIBIOTICS

The long-term, low dose antibiotics have become the primary components in the medical management of reflux, hydronephrosis, and dysfunctional voiding. Prophylactic antibiotics are usually maintained for the first year or until the hydronephrosis resolves. There is no question of biologic plausibility of prophylactic antibiotics in preventing recurrent UTI, however, the adverse effects of antimicrobials and difficulties with long-term adherence to prophylactic strategies are barriers to their effectiveness. Parents who are presented with the option of medical management are excited by the idea of avoiding surgery. However, they are concerned about the extended use of antibiotics and urologists dealing with these families are often questioned about adverse reactions to the medications.

Nitrofurantoin, trimethoprim/sulfamethoxazole (TMP/SMX) are the antibiotics most commonly used in the prevention of UTI, but these medications have adverse reactions in children.[33,34] Common adverse reactions related to nitrofurantoin are gastrointestinal disturbance, cutaneous reactions such as urticaria, maculopapular rash. Adverse reactions related to trimethoprim/sulfamethoxazole are almost exclusively due to the sulfamethoxazole component, most commonly cutaneous reactions. Serious side effects are extremely rare and most are reversible with discontinuation of therapy.[35] Therefore, the long-term use of low dose antibiotics for urinary prophylaxis is mostly safe. Although adverse reactions exist to these medicines, they are much less common in children than in adults, presumably because of the lower dose used for prophylactic therapy and the lack of significant comorbidities and drug interactions in children.

Antibiotic resistance associated with UTI has become an increasingly pressing problem in many countries. Prior studies have shown increasing rate of resistance to commonly used antibiotics.[36,37] The use of antibiotics for prophylaxis also raises the possibility of development of antimicrobial resistance in enteric and oropharyngeal flora.[38] A recent study reported that children receiving prophylactic antibiotic had a high rate of resistance to third-generation cephalosporins.[39] Prudent use of prophylactic antibiotics is suggested to the clinicians.

## CONCLUSIONS

Urinary tract infection in childhood is a common problem and may result in renal scarring which has the potential for diminished renal function and hypertension. Currently, antibiotic prophylaxis is generally recommended for children considered at risk for recurrent UTIs and potential scarring. Although the efficacy and importance of long-term antibiotic prophylaxis has not been tested in well-controlled, prospective studies, the proper use of antibiotic prophylaxis may be of great value in clinical practice, reducing the frequency and clinical expression of UTI. In addition, the common prophylactic antibiotics such as nitrofurnatoin, trimethoprim, and sulfamethoxazole are safe in children for long-term use. Therefore, we believe that long-term antibiotic prophylaxis should be recommenced in infants and children with risk factors, at least until there is evidence that these patients are not endangered by avoiding it.
